# Is there a correlation between inflammatory markers and coagulation parameters in women with advanced ovarian endometriosis?

**DOI:** 10.1186/s12905-019-0860-9

**Published:** 2019-12-30

**Authors:** Shaojie Ding, Qiao Lin, Tianhong Zhu, Tiantian Li, Libo Zhu, Jianzhang Wang, Xinmei Zhang

**Affiliations:** Department of Gynecology, Women’s Hospital, School of Medicine, Zhejiang University, 1 Xueshi Road, Hangzhou Zhejiang, 310006 People’s Republic of China

**Keywords:** Endometriosis, Fibrinogen, Inflammation, Coagulation, Ovarian cyst

## Abstract

**Background:**

Endometriosis is defined as a chronic inflammatory disease. Recent studies have shown that increased coagulation parameters including fibrinogen and platelets are associated with endometriosis. The objective of this study was to determine the levels of inflammatory markers and coagulation parameters and their correlations in women with endometriomas compared to those with benign ovarian cysts or normal pelvic anatomy.

**Methods:**

Between June 2015 and June 2017, a total of 548 women who underwent laparoscopic/laparotomic surgery for ovarian endometriomas (OMA group, *n* = 226), non-endometriosis benign ovarian cysts (Cyst group, *n* = 210) and tubal reanastomosis (Control group, *n* = 112) were recruited in this study. Inflammatory markers including c-reactive protein (CRP), neutrophil-to-lymphocyte ratio (NLR), platelet-to-lymphocyte ratio (PLR) and coagulation parameters including platelet count, thrombin time (TT), prothrombin time (PT), activated partial thromboplastin time, and plasma fibrinogen as well as CA-125 were determined.

**Results:**

Compared with Cyst group and Control group, TT and PT in OMA group were significantly shorter and plasma fibrinogen levels were significantly higher (*P* < 0.05). Moreover, the levels of plasma fibrinogen were positively correlated with CRP, NLR and PLR (*P* < 0.05). In addition, the confidence intervals for the area under the curve (AUC) for CA-125 × fibrinogen were significantly higher than those for CA-125 (0.904–0.952 vs. 0.899–0.949) in the diagnosis of endometrioma.

**Conclusions:**

These results indicate that women with endometriomas demonstrate a hypercoagulable status due to the inflammatory nature of endometriosis. The combined determination for CA-125 and fibrinogen demonstrate a higher area under the curve than the single detection of CA-125 in those with endometriomas compared to these with benign ovarian cysts.

**Trial registration:**

This study was approved by the Human Ethics Committee of the Women’s Hospital, School of Medicine, Zhejiang University (No.20170174) and all women provided written informed consent.

## Background

Endometriosis, defined as the presence of functional endometrial tissue outside the uterine cavity, is considered to be a chronic inflammatory disease, which is related to the increase of pro-inflammatory cytokines and chemokines [1]. The inflammatory process of endometriosis is associated with increased levels of activated macrophages secreted cytokines in the endometriotic lesions and peritoneal fluid (PF), which provides a local microenvironment suitable for the growth and maintenance of endometriosis. The expression levels of interleukin (IL)-1β, IL-6 and tumor necrosis factor (TNF)-α in lesion, endometriotic cyst fluid, and PF have been demonstrated to be increased in women with endometriosis [2–5]. The ratio of neutrophil-to-lymphocyte ratio (NLR) and platelet-to-lymphocyte ratio (PLR) in the peripheral blood of women with endometriosis also increased [6–8]. Moreover, the levels of cytokines [5], free iron, reactive oxygen species [9], matrix metalloproteinases (MMPs) [10], activins [11], and even plasminogen activated system components [12] have also been shown to be elevated in women with ovarian endometriomas compared with other benign ovarian cysts. These different inflammatory and coagulation mediators may identify pathways activated or associated with the development and progression of endometriosis [13].

It has been shown that women with endometriosis appear to be in a hypercoagulable and hyperfibrinolytic status because platelets aggregate in endometriotic lesions [14]. In women with endometriosis, platelet counts (PLT) and plasma fibrinogen levels increase [15, 16], thrombin time (TT) [15] and activated partial thromboplastin time (APTT) decrease [15, 17], while prothrombin time (PT) remains at normal level. Moreover, tissue factor (TF, coagulation factor III) is also increased in the endometriotic lesions and PF in women with endometriosis [14, 18]. TF binds to coagulation factor VIIa, activates coagulation factors IX and X, and induces thrombin formation. Fibrinolysis is a response to elevated levels of coagulation factors and promotes the levels of urokinase-plasminogen activator (u-PA) and plasminogen activator inhibitors (PAIs) in the eutopic endometrium and PF [19, 20].

Inflammatory processes can initiate and promote coagulation, increasing the risk of bleeding, microvascular thrombosis and organ dysfunction [21]. In the coagulation cascade reaction, activated platelets and TF bind to coagulation factors and thrombin to induce inflammation [22, 23]. Activated fibrinogen can also induce thrombin generation, which further activates chemokine production and macrophage adhesion [24].

In this study, we aimed to determine the correlations between coagulation parameters (TT, PT, APTT, and PLT), inflammatory biomarkers [C-reactive protein (CRP), NLR and PLR], compare the differences between patients with endometriomas and patients with non-ovarian endometriosis cysts and without ovarian cysts, and to explore how these parameters can be used as auxiliary biomarkers for diagnosis.

## Methods

### Patients

This study was approved by the Ethics Committee of the Women’s Hospital, Zhejiang University School of Medicine (No.20170174) in accordance with the Declaration of Helsiniki, and we obtained the written informed consent of each participant in this study.

Before the implementation of this study, we used formulas for mismatched case-control studies (α = 0.05, β = 0.1) to determine the size of samples. According to the cut-off of fibrinogen at 2.8 g/L for the diagnosis of endometriomas reported by Chmaj-Wierzchowska et al. [16], P1 and P2 were 0.83 and 0.64 in our small sample test, and the size of samples was at least 111. As a results, a total of 548 women who underwent laparoscopic/laparotomic surgery for ovarian endometriomas (OMA group, *n* = 226), non-endometriosis benign ovarian cysts (Cyst group, *n* = 210) and tubal anastomosis (Control group, *n* = 112) at the Women’s Hospital affiliated to Zhejiang University School of Medicine from June 2015 and June 2017 were recruited in this study. In OMA group, endometriosis was graded according to the Revised American Fertility Society Scoring (rAFS) system (III = 91, IV = 135) [25]. Of the 226 women with endometriosis, 128 (56.6%) women had dysmenorrhea. In Cyst group, 117 (55.7%) women had ovarian mature teratomas, 21 (10.0%) benign ovarian serous tumors, 24 (11.4%) benign ovarian mucinous tumors, 33 (15.7%) simple cysts, 8 (3.8%) follicular cysts, 5 (2.4%) paroophoritic cysts and 2 (1.0%) corpus luteum cysts. In Control group, all women underwent tubal anastomosis because of the requirement of fertility after tubal ligation.

The patients’ demographic and clinical data such as age, gravidity, parity, body mass index (BMI), dysmenorrhea, pelvic pain, and cyst size were extracted and recorded from patients’ original electronic medical record. Due to the lack of women in secretory phase at admission, only women in proliferative phase were included in the study. All patients had no history of hypertension, diabetes, liver and kidney dysfunction, leiomyoma, adenomyosis, and coagulation disorder. No steroid hormone or anticoagulation therapy was given 6 months before surgery.

### Blood assays

Before surgery, all of patients had routine peripheral blood tests including complete blood count, serum biochemistry, coagulation profile, and tumor markers. The neutrophil count, lymphocyte count and PLT were determined using an automatic classification analyzer (Beckman, Coulter LH750). CRP levels were measured with an automatic blood analyzer (Abbott, Architect C16000). Levels of APTT, PT, TT and fibrinogen were determined with an automatic blood coagulation analyzer (STAGO, Evolution ISTA-R-IV, Germany). The levels of serum cancer antigen (CA) 125 were measured by chemiluminescence with an endocrine detector (Roche, COBAS800, France).

### Statistical analysis

Statistical Package for the Social Sciences Statistics v22.0 were used for data analysis. Continuous data were presented as mean ± standard error of the mean (SEM). The comparison of the distribution of continuous data among the three groups was performed with Kruskal–Wallis H test. Mann–Whitney U and χ2 tests were used to compare the medians and frequencies among the groups. Spearman analysis was conducted to determine the correlation among the measured parameters. The optimal cut-off values of the parameters differentiating endometriomas were evaluated with the receiver operating characteristic (ROC) curve analysis. Then, the area under the curve (AUC) was calculated and the sensitivity and specificity of each parameter were determined. *P* values of less than 0.05 (*P* < 0.05) is considered to have statistical significance.

## Results

### Patients’ characteristics

There were no statistical differences with regard to age, body weight, height or BMI between OMA, Cyst and Control groups (*P* > 0.05). Compared with the Cyst and Control groups, the gravidity and parity were significantly lower, and the incidence of dysmenorrhea was significantly higher in the OMA group (P < 0.05). No statistical difference in the size of ovarian cysts between the OMA and Cyst groups was observed (P > 0.05, Table 1).

**Table 1:** Patients’ characteristics

Variable	OMA group	Cyst group	Control group
	(n = 226)	(*n* = 210)	(*n* = 112)
Age (year) ^&^	35.7 ± 0.4	35.9 ± 0.4	35.8 ± 0.5
Height (cm) ^&^	159.2 ± 0.3	159.1 ± 0.3	158.2 ± 0.3
Body weight (kg) ^&^	57.4 ± 0.5	56.6 ± 0.5	57.1 ± 0.6
Body mass index^&^	22.6 ± 0.2	22.3 ± 0.2	22.8 ± 0.2
Gravidity^&&^	2 (0–5)	2 (0–5)^*^	3 (1–5)^***#^
Parity^&&^	1 (0–2)	1 (0–2)^*^	2 (1–2)^***#^
Dysmenorrhea, n (%)	128 (56.6)	26 (12.4) ^***^	8 (7.1)^***^
Cyst size (cm) ^&^	6.2 ± 0.1	6.0 ± 0.2	–

OMA group, women with ovarian endometriomas; Cyst group, women with non-endometriosis benign ovarian cysts; Control group, women undergoing tubal anastomosis; * *P* < 0.05 (compared with OMA group); *** *P* < 0.0001 (compared with OMA group); # *P* < 0.0001 (compared with Cyst group); & mean ± SEM; && median [Range (5–95%)]

### Comparisons of levels of coagulation parameters and inflammatory markers between groups

**Fig. 1:**
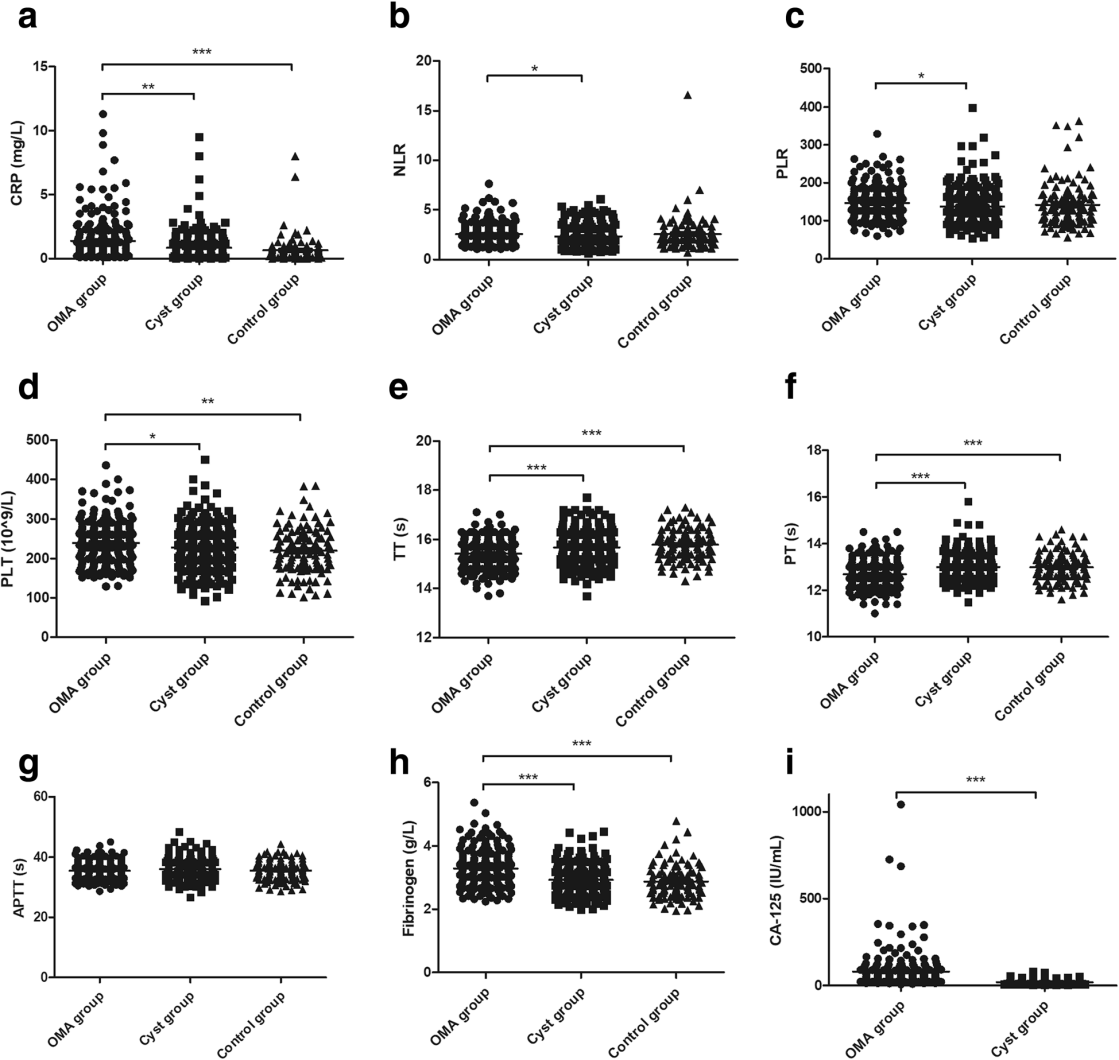
Levels of inflammatory biomarkers and coagulation parameters in groups. **a**-**c**, Inflammatory biomarkers of CRP (**a**), NLR (**b**) and PLR (**c**) were measured among groups. **d**-**i**, Coagulation parameters of PLT (**d**), TT (**e**), PT (**f**), APTT (**g**) and fibrinogen (**h**), as well as cancer antigen CA-125 (**i**) were measured among groups. OMA group, women with ovarian endometriomas; Cyst group, women with non-endometriosis benign ovarian cysts; Control group, women undergoing tubal anastomosis; CRP, C-reactive protein; NLR, neutrophil-to-lymphocyte ratio; PLR; platelet-to-lymphocyte ratio; PLT, platelet count; TT, thrombin time; PT, prothrombin time; APTT, activated partial thromboplastin time;  OMA group;  Cyst group;  Control group* *P* < 0.05; ** *P* < 0.01; *** *P* < 0.0001

The mean (± SEM) levels of CRP (mg/L) were significantly higher in the OMA group (1.38 ± 0.11) than those in the Cyst group (0.86 ± 0.08, *P* < 0.01) and Control group (0.72 ± 0.15, *P* < 0.0001, Fig. 1a). Moreover, the levels of NLR and PLR were significantly higher in the OMA group (2.56 ± 0.07 and 146.4 ± 2.8) than those in the Cyst group (2.34 ± 0.07 and 137.7 ± 3.4, *P* < 0.05; Fig. 1b, c). Furthermore, the levels of PLT (10^9/L) and plasma fibrinogen (g/L) were also significantly higher in the OMA group (239.8 ± 3.6 and 3.29 ± 0.04) than those in the Cyst group (228.4 ± 4.0 and 2.93 ± 0.03, *P* < 0.05) and the Control group (220.0 ± 5.4 and 2.88 ± 0.05, P < 0.05; Fig. 1d, h). However, the time of TT (s) and PT (s) were significantly shorter in the OMA group (15.42 ± 0.04 and 12.69 ± 0.04) than those in the Cyst group (15.68 ± 0.05 and 13.00 ± 0.04, *P* < 0.05) and the Control group(15.78 ± 0.06 and 12.99 ± 0.06, P < 0.05; Fig. 1E–1F). No significant differences with regard to CRP, PLT, fibrinogen, TT or PT between the Cyst and Control groups were found (*P* > 0.05). There were no statistical differences in the APTT between groups (P > 0.05, Fig. 1g).

**Fig. 2:**
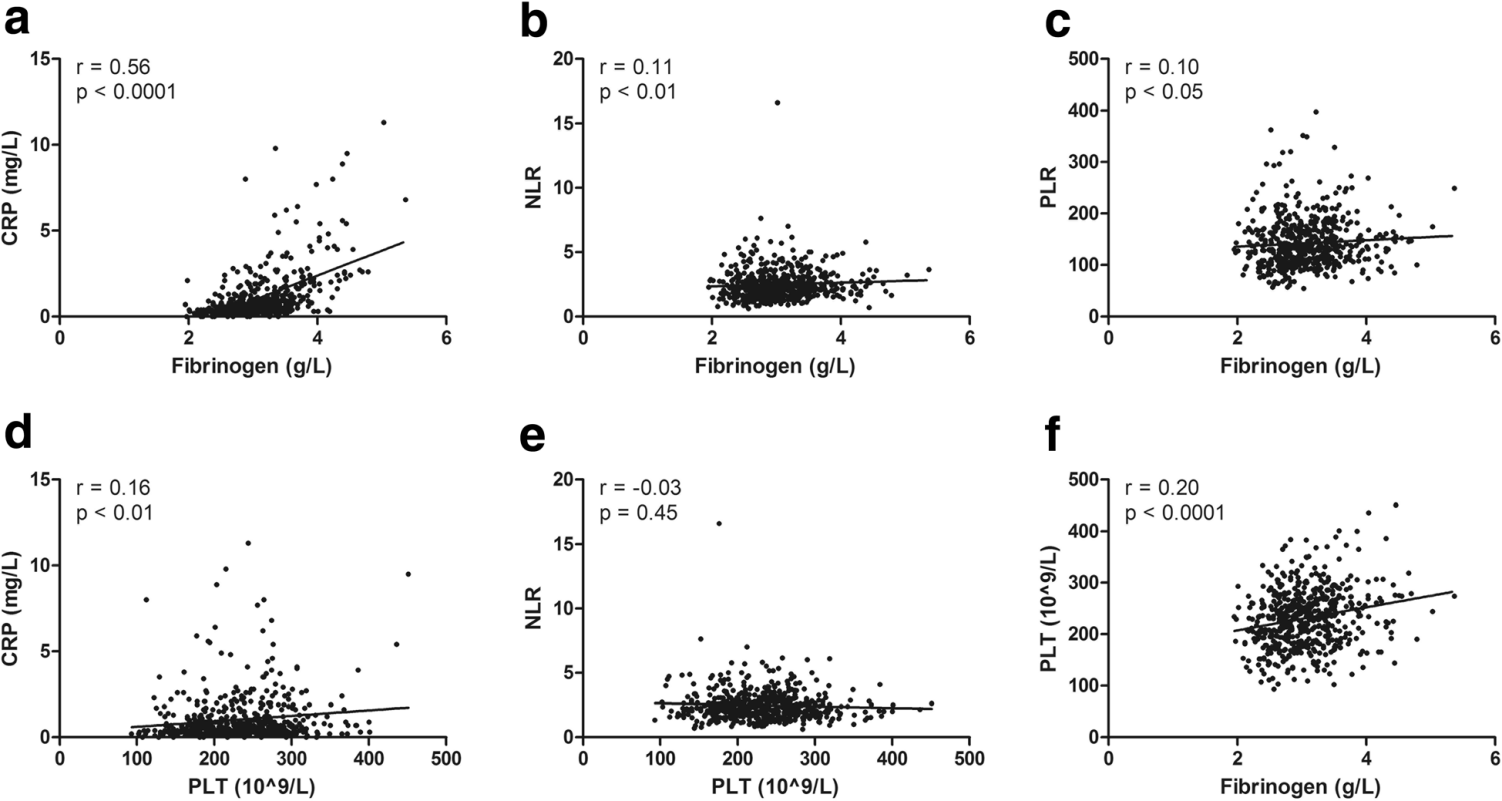
Correlations of coagulation parameters and inflammatory biomarkers. **a**-**c**, The correlations of fibrinogen with CRP (**a**), NLR (**b**) and PLR (**c**) were tested using Spearmen analysis. **d**-**f**, The correlations of PLT with CRP(**d**), NLR (**e**) and fibrinogen (**f**) were tested using Spearmen analysis. CRP, C-reactive protein; NLR, neutrophil-to-lymphocyte ratio; PLR; platelet-to-lymphocyte ratio; PLT, platelet count

Spearman analysis showed that fibrinogen was positively correlated with CRP (r = 0.56, *P* < 0.0001; Fig. 2a), NLR (r = 0.11, *P* < 0.01; Fig. 2b), and PLR (r = 0.10, *P* < 0.05; Fig. 2c). PLT was positively correlated with CRP (r = 0.16, P < 0.01; Fig. 2d) but not significantly correlated with NLR (*P* = 0.45; Fig. 2e). Fibrinogen and PLT demonstrated a significantly positive correlation (r = 0.20, P < 0.0001; Fig. 2f). Moreover, fibrinogen (r = − 0.41, P < 0.0001; r = − 0.37, P < 0.0001 respectively) and PLT (r = − 0.10, *P* < 0.05; r = − 0.19, P < 0.0001, respectively) were negatively correlated with TT and PT but not significantly correlated with APTT (*P* > 0.05).

### Coagulation parameters and inflammatory biomarkers in women with ovarian endometriosis

In the OMA group, no significant correlation between coagulation parameters or inflammatory markers and dysmenorrhea, cyst size or endometriosis stage was found (Table 2). However, the levels of PLT in women with endometriosis who had severe pelvic adhesions were significant higher when compared with women with endometriosis who had mild or no pelvic adhesions (P < 0.05; Table 2). In addition, the time of TT, PT and APTT were shorted, and the levels of fibrinogen were higher in women with endometriosis who had severe pelvic adhesions as compared with women with endometriosis who had mild or no pelvic adhesions, but the differences did not reach statistical significance (P > 0.05; Table 2).

**Table 2:** Coagulation parameters and inflammatory biomarkers in women with ovarian endometriosis

Variables	CRP	NLR	PLR	PLT	TT	PT	APTT	Fb
Dysmenorrhea								
Yes (*n* = 128)	1.46 ± 0.01	2.60 ± 0.10	148.7 ± 3.9	242.3 ± 4.9	15.45 ± 0.05	12.72 ± 0.05	35.86 ± 0.27	3.32 ± 0.05
No (*n* = 98)	1.27 ± 0.02	2.50 ± 0.10	143.3 ± 3.8	236.5 ± 5.2	15.37 ± 0.06	12.65 ± 0.06	35.11 ± 0.28	3.25 ± 0.05
Cyst size, cm								
< = 5 cm (*n* = 66)	1.32 ± 0.02	2.66 ± 0.14	153.2 ± 5.8	237.3 ± 7.3	15.48 ± 0.08	12.71 ± 0.07	35.06 ± 0.34	3.29 ± 0.07
> 5 cm (*n* = 160)	1.40 ± 0.01	2.52 ± 0.08	143.5 ± 3.1	240.8 ± 4.0	15.39 ± 0.04	12.68 ± 0.05	35.73 ± 0.23	3.29 ± 0.04
Stage								
III (*n* = 91)	1.22 ± 0.02	2.58 ± 0.10	145.6 ± 4.5	243.8 ± 5.4	15.38 ± 0.06	12.64 ± 0.06	35.68 ± 0.30	3.24 ± 0.05
IV (*n* = 135)	1.48 ± 0.01	2.54 ± 0.09	146.9 ± 3.5	237.1 ± 4.7	15.44 ± 0.05	12.72 ± 0.05	35.44 ± 0.26	3.32 ± 0.06
Pelvic adhesions^#^								
Yes (*n* = 204)	1.38 ± 0.01	2.52 ± 0.07	147.7 ± 3.0	242.6 ± 3.8*	15.41 ± 0.04	12.68 ± 0.04	35.51 ± 0.20	3.29 ± 0.04
N0 (*n* = 22)	1.37 ± 0.10	2.90 ± 0.32	134.2 ± 6.8	214.0 ± 10.0	15.51 ± 0.13	12.76 ± 0.14	35.77 ± 0.71	3.25 ± 0.11

*CRP* C-reactive-protein (mg/L); *NLR* Neutrophil to lymphocyte ratio; *PLR* Platelet to lymphocyte ratio, *PLT* Platelet count (10^9/L), *TT* thrombin time (s); *PT* (s), Prothrombin time, *APTT* Activated partial thromboplastin time (s), *Fb* Fibrinogen (g/L). #, Endometriosis women with severe pelvic adhesions; * *P* < 0.05 (compared with no severe pelvic adhesions)

### Diagnostic value of CA-125, coagulation and inflammatory parameters in ovarian endometrioma

**Fig. 3:**
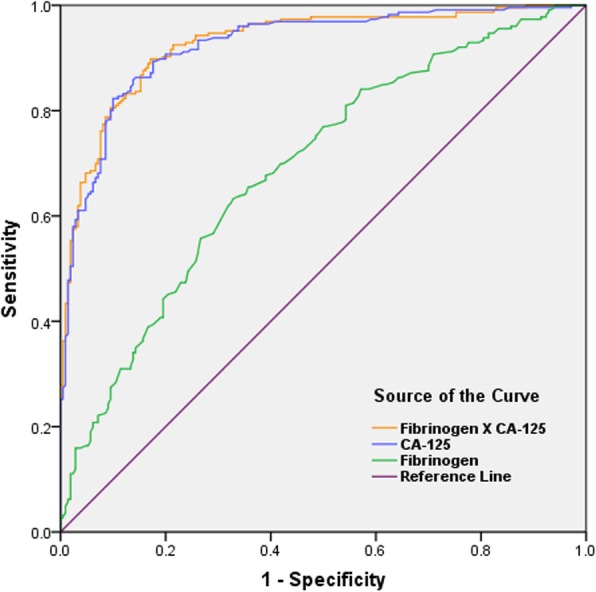
Receiver–operating characteristic curves of CA-125, fibrinogen, and the combined marker for the diagnosis of endometriomas

The levels of CA-125 in OMA group were significantly higher than those in Cyst group (80.0 ± 7.1 vs. 19.2 ± 0.8; *P* < 0.0001; Fig. 1i), with a cut-off value at 30.75 IU/mL. The AUC of CA-125 was 0.924 (95% confidence interval: 0.899–0.949) with sensitivity and specificity reaching 82.3 and 90.0%, respectively (Table 3). The optimal cut-off points for inflammatory markers, coagulation parameters, and the combined marker (plus CA-125) for determining endometriosis were evaluated by ROC analysis (Table 3). The results also showed that the sensitivity and specificity of using any of these factors alone for diagnosis of endometrioma were lower than those of CA-125. For fibrinogen, the cut-off value was 3.09 g/L with a validity power at 0.9998 (P1 = 0.63, P2 = 0.28, β = 0.0002) in present study. However, the combination of CA-125 and fibrinogen showed the highest AUC of 0.928 (0.904–0.952) with sensitivity of 0.898 and specificity of 0.829 (Fig. 3). The diagnostic effects of TT or PT combined with CA-125 were also better than that of CA-125 alone. (Table 3).

**Table 3:** The diagnostic value of CA-125, coagulation and inflammatory parameters in ovarian endometrioma

Parameters	AUC (95% CI)	Sensitivity (%)	Specificity (%)	Cutoff value
CA-125 (IU/mL)	0.924 (0.899–0.949)	82.3	90.0	30.75
CRP (mg/L)	0.630 (0.578–0.682)	78.8	40.1	0.35
NLR	0.575 (0.522–0.629)	77	39.5	1.88
PLR	0.584 (0.530–0.638)	65.9	51.4	128.3
PLT (10^9/L)	0.557 (0.503–0.611)	41.2	69.5	253.5
TT (s)	0.613 (0.560–0.665)	69.5	49.1	15.35
PT (s)	0.643 (0.592–0.695)	56.7	65	12.85
APTT (s)	0.546 (0.492–0.600)	33.3	76.1	37.45
Fibrinogen (g/L)	0.692 (0.643–0.741)	63.3	67.1	3.09
CA-125 * CRP (IU*mg/mL*L)	0.832 (0.794–0.869)	69.5	82.6	24.17
CA-125 * NLR (IU/mL)	0.899 (0.870–0.927)	84.1	81.0	60.06
CA-125 * PLR (IU/mL)	0.907 (0.879–0.935)	88.1	80.0	3383.16
CA-125 * PLT (IU*10^9/mL*L)	0.909 (0.882–0.937)	80.1	87.6	6695.85
CA-125 / TT (IU*s/mL)	0.926 (0.902–0.951)	85.0	88.1	1.84
CA-125 / PT (IU*s/mL)	0.927 (0.903–0.952)	87.2	85.7	2.06
CA-125 / APTT (IU*s/mL)	0.924 (0.899–0.949)	88.1	84.3	0.75
CA-125*Fibrinogen(IU*g/mL*L)	0.928 (0.904–0.952)	89.8	82.9	73.77

*AUC* area under the curve, *CRP* C-reactive protein, *NLR* neutrophil-to-lymphocyte ratio, *PLR* platelet-to-lymphocyte ratio, *PLT* platelet count, *TT* thrombin time, *PT* prothrombin time, *APTT* activated partial thromboplastin time

## Discussion

Women with ovarian endometriomas demonstrated a hypercoagulable and inflammatory status, based on increased levels of CRP, PLT, and fibrinogen as well as shortened TT and PT.

Evidence shows that systemic inflammation activates the coagulation system in response to TF-mediated thrombin generation [26]. TF can be secreted by activated monocytes and endothelial and polymorphonuclear cells, which are regulated by TNF-α, IL-1β, and lipopolysaccharide [27–29]. Ding et al. have [14] reported that TF concentrations are significantly elevated in primary endometriotic stromal cells. TF binds to circulating factor VIIa to mediate the activation of factors IX and X and generates thrombin [30]. It has been reported in endometriotic stromal cells, thrombin and proteinase-activated receptor (PAR)-1 agonist induce IL-6 and IL-8 secretion and cell proliferation [23]. Thrombin also can binds to another type of PAR expressed in endometriotic stromal cells, PAR-2, mediating the production of chemokines and cytokines such as IL-8, monocyte chemotactic protein-1, MMP-2, and cyclooxygenase-2 [22].

Studies have reported that inflammatory also induces fibrinolysis activation in endometriosis. Plasmin, an active enzyme, can degrade various extracellular matrix proteins and activate MMPs [31]. The eutopic endometrium of women with ovarian endometriosis has been shown to express high levels of MMP-3, which can hydrolyze and inactivate PAI-1, regulating cell-associated plasmin activities [32]. Higher levels of PAI-1 and tissue inhibitor of metalloproteinase-1 in ovarian endometriomas prevent endometriotic cysts from invading surrounding ovarian tissues [33, 34]. Meanwhile, activated plasmin may induce expression of proinflammatory cytokines such as IL-1α, IL-1β, TNF-α, and TF [28]. Inflammatory changes and activated fibrinolytic systems in women with endometriomas may play an important role in the development and progression of endometriosis.

Fibrinogen influences thrombin formation, platelet aggregation, blood rheology and blood viscosity. Fibrinogen levels are elevated in a variety of diseases such as diabetes and nephrotic diseases, and are associated with an increased risk of cardiovascular disease [35, 36]. Fibrinogen is closely associated with hypercoagulation. Kurata et al. [37] reported that TT, APTT, and PT were all significantly shortened in canines injected with fibrinogen. In the present study, the levels of plasma fibrinogen were significantly higher in women with ovarian endometriomas than those in women with non-endometriosis benign ovarian cysts and those in women without ovarian cysts. These results are in agreement with those of previous reports [15, 16]. We also found that TT and PT were significantly shortened in patients with endometrioma, but there was no difference in APTT. In coagulation cascade, the procoagulation factor, PT, measures the extrinsic coagulation pathway. PT is most sensitive to factor VII (FVII) levels as the latter exhibits a short half-life [38]. Given PT is initiated by TF, our findings correspond to previous studies that have reported the elevation of TF in endometriotic lesions and PF in women with endometriosis [14, 18]. However, Paola et al. [17] demonstrated shortened APTT and constant TT, whereas Guo et al. [15] reported shortened TT and APTT and constant PT in women with endometriosis. These different results may be attributed to the different sample sizes, conditions and techniques applied between the studies, considering that blood assays are highly dependent on the combination of reagents and instruments. For coagulation parameters, specific reagents and different manufacturers usually lead to variable results [38]. Obviously, the coagulation function of women with endometriosis needs further study.

CA-125 is a marker and often used in the diagnosis of endometriomas. Some studies reported that NLR as an adjunct to CA-125 is a useful diagnostic marker [6, 7]. However, some studies refute this claim because NLR has not yet been fully investigated and is not suitable as a diagnostic tool for advanced endometriosis [39, 40]. In our study, we demonstrated that the coagulation factors TT, PT, and fibrinogen were more reliable as complementary auxiliary markers of CA-125 for identifying ovarian endometrioma from non-endometriosis benign ovarian cysts. Obviously, the discrepancies among the studies can be attributed to the differences in sample size, experimental measurement methods, and instruments used.

The primary limitation of our study is its retrospective design. All the recruited women were in proliferative phase of menstrual cycle. The coagulation stability in women with endometriosis remains unknown while coagulation status is usually unaffected by menstrual cycle in healthy women [41]. Moreover, only women with advanced endometriosis (stages III–IV) were included in the study. Therefore, the coagulation function and its influence on inflammation in women with early-stage endometriosis are still uncertain.

A combined influence of inflammatory response and hypercoagulation status may exist in advanced stages of endometriosis. At present, the treatment of endometriosis often focuses on anti-inflammatory control. Modulation of the coagulation pathway in endometriosis may provide another potential treatment option. Studies have reported inhibition of inflammation by blocking formation of TF-PAR-2 and the TF-VIIa signaling pathway [42]. In a mouse model of endometriosis, a chimeric immunoconjugate molecule specifically targeting endothelial TF in ectopic implants has been shown to obliterate the endometriotic implant by vascular disruption without reducing fertility [43]. Guo et al. [44] reported that targeting P-selectin-mediated platelet adhesion can reduce the size of endometriotic lesions, improving general hyperalgesia and resulting in reduction of macrophage infiltration and fibrotic tissue content. Thus, in order to determine whether abnormal coagulation parameters contribute to the diagnosis and treatment of endometriosis, a more detailed study of large samples is needed.

In conclusion, our findings suggest that women with ovarian endometriomas demonstrate a hypercoagulable status potentially attributable to the inflammatory nature of endometrioma. Plasma fibrinogen is an auxiliary marker of serum CA-125 in the diagnosis of endometriosis.

## Data Availability

The datasets used and/or analyzed during the current study are available from the corresponding author on reasonable request.

## Abbreviations

APTT: Activated partial thromboplastin time
AUC: Area under the curve
BMI: Body mass index
CA: Cancer antigen
CRP: C-reactive protein
IL: Interleukin
MMP: Matrix metalloproteinase
NLR: Neutrophil-to-lymphocyte ratio
PAIs: Plasminogen activator inhibitors
PAR: Proteinase-activated receptor
PF: Peritoneal fluid
PLR: Platelet-to-lymphocyte ratio
PLT: Platelet count
PT: Prothrombin time
ROC: Receiver operating characteristic
SEM: Standard error of the mean
TF: Tissue factor
TNF: Tumor necrosis factor
TT: Thrombin time
uPA: urokinase plasminogen activator
